# Near-infrared spectroscopy–guided personalized repetitive transcranial magnetic stimulation for bipolar depression: a case report

**DOI:** 10.3389/fpsyt.2024.1514153

**Published:** 2025-01-20

**Authors:** Chun-Hung Chang, Wen-Chun Liu, Po-Han Chou

**Affiliations:** ^1^ An Nan Hospital, China Medical University, Tainan, Taiwan; ^2^ College of Medicine, China Medical University, Taichung, Taiwan; ^3^ Shu-Zen Junior College of Medicine and Management, Kaohsiung, Taiwan; ^4^ Department of Nursing, National Tainan Junior College of Nursing, Tainan, Taiwan; ^5^ Department of Brain Reserch, Brain Mental Health Clinic, Zubei, Taiwan

**Keywords:** near-infrared spectroscopy, NIRS, transcranial magnetic stimulation, depression, precision medicine, bipolar depression, rTMS

## Abstract

**Introduction:**

Transcranial magnetic stimulation (TMS) is a common treatment for depression, particularly in patients unresponsive to conventional therapies. High-frequency (10 Hz), low-frequency (1 Hz), or bilateral (left, high-frequency; right, low-frequency) stimulation of the dorsolateral prefrontal cortex (DLPFC) has been demonstrated to be effective in studies based on prefrontal asymmetry theory, which suggests that depression is associated with reduced left frontal function and increased right frontal function. However, few reliable predictors or biomarkers are available for personalizing treatment protocols on the basis of a patient’s brain function. Near-infrared spectroscopy (NIRS), a noninvasive neuroimaging tool that assesses functional changes in the brain during cognitive tasks, can measure a patient’s bilateral frontal lobe function in real time. Thus, this tool can aid the development of personalized TMS protocols for patients with depression.

**Methods:**

A 19-year-old woman presented to our psychiatric clinic with bipolar depression. NIRS was performed to select an appropriate TMS protocol for the patient. A verbal fluency test revealed bilateral low frontal lobe function. Thus, we selected a TMS protocol involving 10 sessions of bilateral high-frequency stimulation over 4 days, with each session delivering 3000 pulses on each side of the DLPFC.

**Results:**

Before treatment, the patient’s scores on the Hamilton Depression Rating Scale (HAMD), Beck Depression Inventory (BDI), Beck Anxiety Inventory (BAI), and Young Mania Rating Scale were 40, 57, 40, and 6, respectively. After treatment, her depressive symptoms substantially improved, with HAMD, BDI, and BAI scores decreasing to 17, 21, and 14, respectively. Although the treatment led to side effects such as dizziness and headache, these effects resolved after the treatment. At the 6-month follow-up, the patient’s condition was still stable, with HAMD, BDI, and BAI scores of 10, 13, and 7, respectively.

**Conclusion:**

Our case suggests that NIRS can guide the selection of appropriate TMS protocols for patients with bipolar depression. Although our findings are promising, further randomized controlled trials are needed to validate the efficacy and safety of and determine the optimal parameters for this approach.

## Introduction

Transcranial magnetic stimulation (TMS) has transformed the treatment landscape for depression, particularly for patients unresponsive to conventional therapy. TMS is a noninvasive technique that involves the use of magnetic fields to stimulate nerve cells in the brain, specifically those in brain areas involved in mood regulation—for example, the dorsolateral prefrontal cortex (DLPFC) (1, 2). This technique has been approved by the US Food and Drug Administration for treating depression; it offers a promising alternative for many patients because it can be administered in an outpatient setting, has minimal side effects, and is compatible with other treatments (3, 4). Despite advancements, the overall efficacy of current therapies remains limited. A randomized controlled trial reported that the rate of patient response to standard sequential repetitive TMS (rTMS) was <50% (5). The response rate varies partly because of differences in response patterns among patients with major depressive disorder (MDD) receiving rTMS. In their study involving 388 patients with MDD who were treated with rTMS or intermittent theta-burst stimulation (iTBS), Kaster et al. identified the following four response patterns (6, 7): nonresponse (11%), rapid response (19%), linear response with moderate-to-severe initial symptoms (30%), and linear response with mild initial symptoms (40%). Marked interpersonal differences in response rate become evident from the first week of treatment, highlighting the importance of personalized rTMS strategies and the need for biomarkers that reliably predict treatment outcomes (6, 7).

Near-infrared spectroscopy (NIRS) is an advanced, noninvasive functional neuroimaging technique that assesses neural activity in the brain’s bilateral frontotemporal regions in real time. This technique has several advantages over traditional imaging methods such as positron emission tomography, single photon emission computed tomography, and functional magnetic resonance imaging (8). NIRS can help measure the levels of oxyhemoglobin and deoxyhemoglobin at the bedside. These measurements indicate local cerebral blood volume and are strongly correlated with functional magnetic resonance imaging signals (9, 10). Some studies suggests that NIRS can help predict the rTMS response of patients with MDD (6, 9).

The development of accelerated TMS protocols represents a key advancement in the treatment of depression (3, 11). Traditional TMS protocols necessitate daily sessions conducted over several weeks, which may be time-prohibitive for some patients. However, accelerated TMS condenses the treatment into a relatively short time frame by facilitating multiple sessions per day or over consecutive days. This approach leads to rapid symptom relief—an essential treatment goal for patients with severe or conventional therapy–resistant depression. In the treatment of depression, bilateral DLPFC stimulation is more effective than unilateral DLPFC stimulation (12, 13). Kazemi et al. demonstrated that bilateral rTMS markedly improved executive function, enhanced verbal memory, and alleviated depressive symptoms in patients with bipolar depression (14).

The present case report explores the benefits of integrating NIRS with TMS in the treatment of bipolar depression. Specifically, NIRS was used to guide the selection of an appropriate protocol for accelerated, bilateral, high-frequency DLPFC stimulation. This report provides key insights into effective and rapid treatment approaches for patients with severe depression. Our findings may inform personalized, biomarker-guided therapeutic protocols for bipolar depression.

## Case report

A 19-year-old woman presented to our psychiatric clinic with severe depression after a suicide attempt. Her depressive symptoms had first emerged during middle school. The disease was characterized by declining academic performance (her rank dropped from the top 100 out of 800 students to lower than 400), social phobia (to the point where she needed classmates’ assistance for basic tasks such as responding to a roll call), and self-harm behaviors (cutting her wrists with sharp objects). The patient was also cyberbullied by her peers. In addition to a depressed mood, she exhibited symptoms such as anhedonia, feelings of worthlessness, psychomotor retardation, and strong suicidal ideation. Her family sought help from a psychiatric clinic, where she received a diagnosis of MDD initially. She was treated with escitalopram (started at 10 mg/day and increased to 20 mg/day over 1 month) and psychotherapy. Over time, her symptoms ameliorated, allowing her to graduate from high school and enroll in university.

However, the patient’s depression worsened 2 months before visiting our brain stimulation center. The aforementioned clinic had increased her antidepressant dosage, and she developed manic symptoms such as overexcitement, talkativeness, and irritability. After her medications were adjusted, her depression relapsed, and she again had suicidal thoughts (repeatedly contemplating jumping from a window and struggling to resist the impulse). Bipolar depression was suspected on the basis of the criteria outlined in *Diagnostic and Statistical Manual of Mental Disorders*, *Fifth Edition*. The treatment regimen involved lurasidone (20 mg/day), duloxetine (60 mg/day), quetiapine (200 mg/day) + lamotrigine (50 mg/day), and psychotherapy for 4 weeks. Unfortunately, she did not respond well to these medications and experienced side effects such as dizziness, nausea, and weight gain. Because of her unresponsiveness, her family decided to opt for rTMS. Thus, she was referred to our brain stimulation center for consultation. Notably, the patient had a suspected family history of psychiatric illness: her grandmother’s sister might have had a relevant condition.

The Beck Depression Inventory (BDI) and Hamilton Depression Rating Scale (HAMD) were administered to determine the severity of her depression. In addition, the Beck Anxiety Inventory (BAI) was administered to determine the severity of anxiety. Total scores on the BDI and BAI range from 0 to 63, with higher scores indicating more severe symptoms. Furthermore, the Young Mania Rating Scale was administered to determine the severity of mania. Before treatment, the patient’s scores on the HAMD, BDI, BAI, and Young Mania Rating Scale (YMRS) were 40, 57, 40, and 6, respectively.

**Figure 1:**
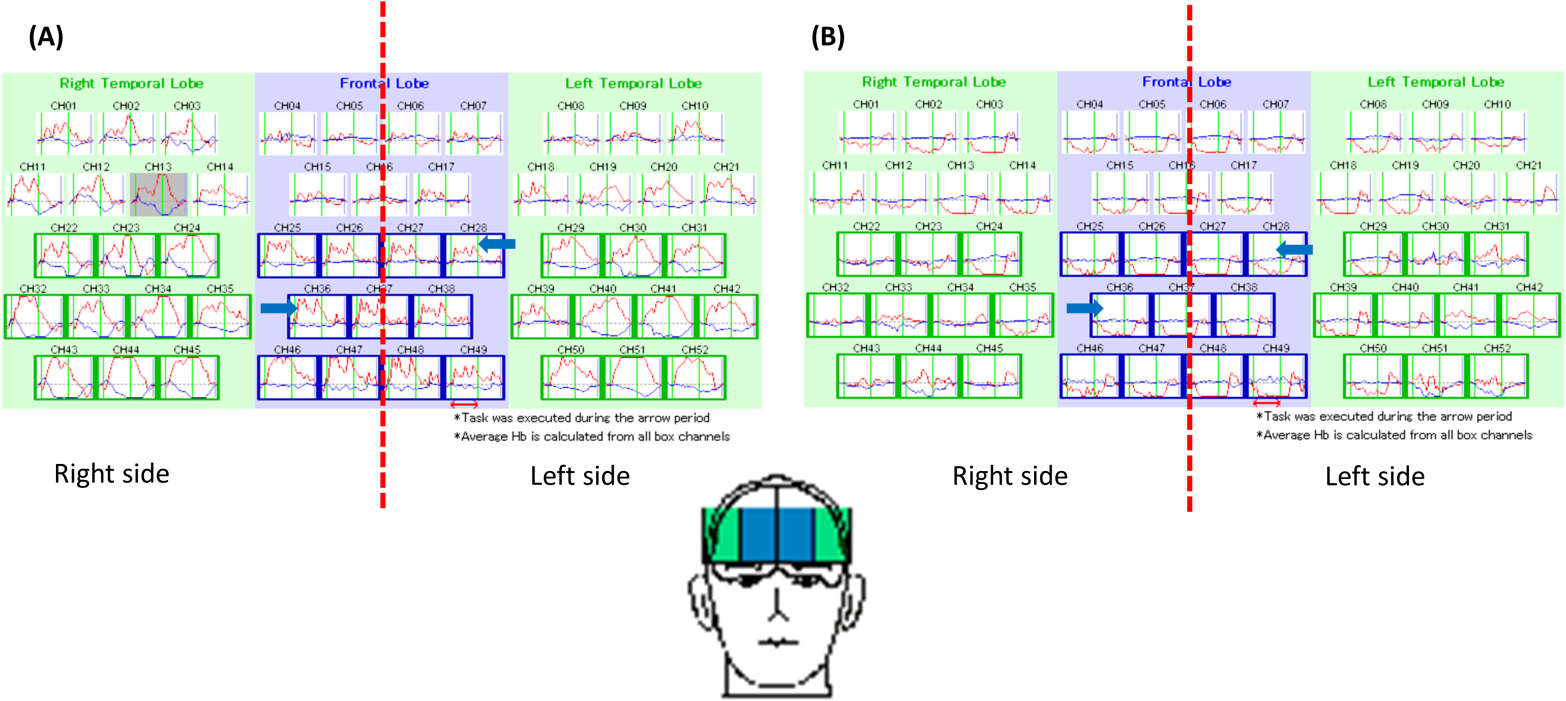
This figure demonstrated the pattern of brain activity during a verbal fluency test in a healthy individual **(A)** and our patient **(B)**. As indicated by blue arrows, the frontal lobe activity in the patient was obviously lower than a healthy individual.

After obtaining informed consent from the patient, we performed NIRS to precisely assess her frontal lobe function during a verbal fluency test. The NIRS results revealed bilateral hypofunction in the DLPFC (**Figure 1**). Thus, high-frequency rTMS of the bilateral DLPFC was initiated. The treatment was delivered using an Apollo TMS Therapy stimulator (MAG & More, Germany) equipped with a figure-of-eight coil. Coil localization was based on an algorithm developed by Beam et al. (15), with the Beam-F3 and Beam-F4 positions used for the left DLPFC and right DLPFC, respectively. The treatment protocol was as follows: 120% motor threshold, 10-Hz frequency, 4-s trains, 75 trains per session, and a total of 6000 pulses per session. We first targeted the left DLPFC and then the right DLPFC. Because the patient’s family wanted her to rapidly recover and return to university, we performed accelerated bilateral stimulation, which was delivered in 10 sessions over 4 days (2 to 3 sessions per day).

After this treatment, the patient’s depressive symptoms gradually ameliorated, with her HAMD, BDI, BAI, and YMRS scores were 17, 21, 14, and 8 respectively. Although the treatment led to side effects such as dizziness and headache, they resolved after the treatment. At the 6-month follow-up, the patient’s condition was still stable, and she had HAMD, BDI, BAI, and YMRS scores of 10, 13, 7, and 6, respectively (**Table 1**).

**Table 1:** Severity of anxiety and depression before and after TMS.

Symptoms	Before TMS	After TMS	6-month follow-up
HAMD	40	17	10
BDI	57	21	13
BAI	40	14	7
YMRS	6	8	6

TMS, transcranial magnetic stimulation; HAMD, Hamilton Depression Rating Scale; BDI, Beck Depression Inventory; BAI, Beck Anxiety Inventory; YMRS, Young Mania Rating Scale.

## Discussion

To the best of our knowledge, this case report is the first to suggest that NIRS-guided personalized TMS is effective for patients with bipolar depression who are unresponsive to conventional therapy. NIRS facilitates the diagnosis and treatment of psychiatric disorders by measuring brain activity (16–18). In the treatment of mood disorders, bilateral high-frequency stimulation of the DLPFC is more effective than are traditional unilateral approaches (5). Our patient exhibited substantial posttreatment improvements in depression scores, and the treatment had minimal side effects. Therefore, our approach can effectively treat patients with severe depression who are unresponsive to conventional therapy.

We opted for an rTMS protocol that was different from conventional approaches such as high-frequency, low-frequency, or bilateral (left, high-frequency; right, low-frequency) stimulation of the DLPFC. Because NIRS revealed bilateral hypofunction in the patient’s frontal region, we selected high-frequency stimulation to improve bilateral frontal lobe function. Traditional rTMS protocols for treating depression are typically based on prefrontal asymmetry theory, which links depression with relative hypoactivity of the left DLPFC and hyperactivity of the right DLPFC (19). However, this finding is not consistent across all patients with depression (20, 21). For instance, Li et al. found bilateral hypofrontality in patients with medication-resistant depression (22), a finding that aligns with ours. Furthermore, Yan et al. demonstrated that high-frequency stimulation of the right DLPFC mitigated depressive symptoms in patients with posttraumatic stress disorder (23). Therefore, our rTMS protocol involving high-frequency stimulation of the bilateral frontal lobes is safe for patients with depressive symptoms.

As mentioned, bilateral DLPFC stimulation may treat depression more effectively than does unilateral DLPFC stimulation. A study was conducted with 30 patients diagnosed with bipolar depression, who were randomly assigned to one of two treatment groups. The bilateral group (n = 15) received rTMS to the left DLPFC at 10 Hz and the right DLPFC at 1 Hz. In contrast, the unilateral group (n = 15) received rTMS to the right DLPFC at 1 Hz. Participants in both groups underwent 20 treatment sessions. The response rate in the bilateral stimulation group was significantly higher compared to the unilateral group (80% vs. 47%) (24). A recent network meta-analysis showed that bilateral rTMS outperformed sham treatment in terms of treatment response (RR = 2.08, 95% CI: 1.01–4.27), when compared to other unilateral rTMS interventions (25). In a randomized, sham-controlled trial, 60 patients with treatment-resistant, recurrent MDD were divided into four groups (n = 15 per group). Group A received continuous theta-burst stimulation (TBS) of the right DLPFC, Group B received iTBS of the left DLPFC, Group C received bilateral stimulation (continuous TBS of the right DLPFC and iTBS of the left DLPFC), and Group D received sham TBS. After 2 weeks of treatment, bilateral DLPFC stimulation (Group C) proved more effective than unilateral stimulation (Groups A and B). The patients’ HAMD scores decreased by 22.5% in Group A, 42.3% in Group B, and 52.5% in Group C (*p* = 0.001). Furthermore, the response rate was significantly higher for bilateral stimulation than for unilateral stimulation (66.7% [Group C] vs. 25.0% [Group A] and 40.0% [Group B]; *p* = 0.01) (12).

In the study conducted by Li et al., patients received bilateral TBS (12). We selected bilateral 10-Hz stimulation for our patient for three key reasons. First, NIRS indicated low activity in the bilateral DLPFC, prompting us to select high-frequency stimulation to activate the DLPFC. Second, evidence suggests no significant difference in antidepressant efficacy between TBS and 10-Hz stimulation (26). However, a study involving 30 patients with MDD reported that 10-Hz rTMS of the left DLPFC for 3 consecutive weeks more effectively enhanced long-term potentiation–like plasticity and mitigated clinical symptoms than did iTBS (27). Another study that randomly allocated 41 patients with poststroke cognitive impairment to receive either high-frequency (5-Hz) rTMS (n = 11), iTBS (n = 15), or sham stimulation (n = 15) indicated that the high-frequency rTMS was more effective than iTBS in modulating attention (*p* = 0.016) (28). Finally, 10-Hz rTMS may be more effective in women than in men. A study involving 414 patients with MDD who were treated with either 10-Hz rTMS or iTBS revealed sex-based differences in treatment response (29). *Post hoc t* tests indicated that the improvements at sessions 10 and 30 were significantly greater for male patients receiving iTBS than for female patients receiving iTBS (difference between male and female patients [Δ] = 9% [*p* = 0.041] and 14% [*p* = 0.035] for sessions 10 and 30, respectively). At session 10, a trend toward greater improvement was noted among male patients receiving iTBS than among those receiving 10-Hz rTMS (Δ = 7%; *p* = 0.068); however, this trend was not observed at session 30. By contrast, at both sessions 10 and 30, female patients receiving 10-Hz rTMS exhibited greater improvements than did those receiving iTBS (Δ = 6% [*p* = 0.11] and 7% [*p* = 0.15] for sessions 10 and 30, respectively). At session 10, female patients receiving 10-Hz rTMS exhibited slightly greater improvements than did male patients (Δ = 4%; *p* = 0.12).

As mentioned, this case report is the first to report the use of NIRS to guide accelerated bilateral DLPFC stimulation in a patient with bipolar depression. Although our report provides valuable insights, it has some limitations. First, the absence of a control group makes it challenging to attribute the observed improvements solely to the intervention. Without comparing treatment outcomes for the new approach versus standard care or placebo, we cannot determine the efficacy of our approach or the generalizability of our findings. Second, the focus on a single patient limited our ability to draw broad conclusions. Because of the unique circumstances and personal attributes of the patient, our findings cannot be extrapolated to the wider population receiving similar treatments. Finally, although accelerated bilateral stimulation appears to be promising, its long-term outcomes and potential risks remain unknown. In the future, large-scale randomized controlled trials should be conducted to establish its safety and efficacy profiles and identify its optimal parameters. The use of NIRS biomarkers to assess outcomes following rTMS treatment, along with long-term follow-up, is warranted.

## Conclusion

This case report offers valuable insights into the treatment of bipolar depression with NIRS-guided personalized rTMS. Our findings highlight the potential of using NIRS to real time monitor prefrontal function and guiding selection of rTMS protocol when treating patients. Nonetheless, further studies, particularly randomized controlled trials, are needed to validate the efficacy and safety of our approach and determine its optimal parameters.

## Data Availability

The original contributions presented in the study are included in the article/supplementary material. Further inquiries can be directed to the corresponding author.
